# Supplementary material for: Regional Analysis of Associations between Infant and Young Child Feeding Practices and Diarrhoea in Indian Children
Source: Int J Environ Res Public Health. 2020 Jul 1;17(13):4740. doi: 10.3390/ijerph17134740 (PMC7370018; doi:10.3390/ijerph17134740)
Supplement: Supplementary file 1 [file ijerph-17-04740-s001.pdf]

Supplementary Table 1 - Characteristics of the study population (*N*= 90596), NFHS-4

Characteristics	North (*N* = 11200)		South (*N*= 16469)		East (*N* = 23317)		West (*N* = 11512)		Central (N = 24870)		North-East (*N* = 3228)	
	N†	%	*N*	%	*N*	%	*N*	%	*N*	%	*N*	%
Child characteristics												
**Sex of child**												
Male	6014	53.6	8544	51.9	12183	52.3	5972	51.9	13139	52.8	1652	51.2
Female	5186	46.3	7924	48.2	11134	47.7	5540	48.1	11730	47.2	1576	48.8
**Received vaccinations**												
No	646	5.8	593	3.6	1350	5.8	924	8.0	2036	8.2	495	15.3
Yes	10553	94.2	15876	96.4	21968	94.2	10588	92.0	22834	91.8	2733	84.7
**Birth order**												
1	4377	39.1	7153	43.4	8485	36.4	4776	41.5	8204	32.9	1301	40.3
2 – 4	6170	55.1	9170	55.7	12995	55.7	6458	56.1	14092	56.7	1684	52.1
≥ 5	653	5.8	146	0.9	1837	7.9	277	2.4	2573	10.4	244	7.5
**Size of baby**												
Small	1183	10.7	1376	8.4	3009	13.1	1364	11.9	3620	14.7	429	14.2

Average	8394	75.9	10211	62.3	15124	65.7	7381	64.5	17677	71.8	1976	65.3
Large	1478	13.3	4789	29.2	4887	21.2	2706	23.6	3303	13.4	622	20.5
Maternal characteristics												
**Mother's age (years)**												
15-19	389	3.5	910	5.5	2066	8.9	707	6.1	799	3.2	276	8.5
20-34	10223	91.3	15096	91.6	19816	84.9	10398	90.3	22441	90.2	2646	81.9
35-49	588	5.2	463	2.8	1435	6.2	407	4.0	1629	6.5	307	9.5
**Mother's Education**												
No education	3165	28.2	1815	11	8731	37.4	1529	13.3	8512	34.2	625	19.4
Primary	1580	14.1	1368	8.3	3420	14.7	1366	11.8	3833	15.4	546	16.9
Secondary	6455	57.6	13286	80.7	11167	47.9	8617	74.9	12525	50.4	2058	63.7
**Mother's Marital Status**												
Currently married	11142	99.5	16353	99.3	23158	99.5	11412	99.2	24689	99.3	3176	98.5
Formerly married	48	0.4	111	0.7	126	0.5	85	0.7	163	0.7	47	1.5
**Mother's Employment**												
Did not	1775	89.5	2809	88.4	3438	90.4	2025	85	3539	87.8	478	90.3

Worked	207	10.4	365	11.5	363	9.5	357	15	493	12.2	51	9.7
**Mother's Religion**												
Hindu	8394	74.9	13444	81.6	17977	77.1	9134	79.3	20795	83.6	1508	46.71
Muslim	1685	15	2059	12.5	4606	19.8	1611	14	3953	15.9	1050	32.53
Christianity	1121	10	965	5.8	734	3.1	767	6.7	121	0.5	670	20.75
Family/household characteristics												
**Wealth index**												
Poor	2926	26.1	3335	20.2	16742	71.8	3220	27.9	13644	54.9	2060	63.8
Middle	2209	19.7	4802	29.1	3514	15.1	2779	24.1	4430	17.8	636	19.7
Rich	6065	54.1	8332	50.5	3061	13.1	5512	47.8	6795	27.3	533	16.5
**Type of caste or tribe**												
Schedule caste	2865	25.5	3818	23.1	5256	22.5	1803	15.7	5568	22.3	282	8.7
Schedule tribe	1021	9.1	981	5.9	2515	10.8	1808	15.7	2475	9.9	911	28.2
Other backward castes	4142	36.9	9207	55.9	9759	41.8	3543	30.8	12531	50.3	617	19.1
Others‡	3172	28.3	2463	14.9	5787	24.8	4358	37.8	4295	17.3	1418	43.9
Media factors												

**Watches television**												
No	2288	20.4	1057	6.4	11354	48.7	1940	16.8	9451	38	1196	37
Yes	8912	79.6	15412	93.6	11963	51.3	9572	83.2	15419	62	2033	63
**Reads newspaper**												
No	6920	61.8	7923	48.1	18150	77.8	6532	56.8	18109	72.8	2390	74
Yes	4280	38.2	8545	51.9	5167	22.2	4979	43.2	6761	27.2	838	26
**Listening radio**												
No	9766	87.2	13990	84.9	20310	87.1	10009	86.9	21666	87.1	2791	86.4
Yes	1434	12.8	2479	15.1	3007	12.9	1502	13.1	3203	12.9	438	13.6
Health service characteristics												
**Antenatal clinical visits**												
None	1269	11.3	1069	6.5	6083	26.1	1105	9.6	4826	19.4	469	14.5
1 – 3	4127	36.8	2493	15.1	7954	34.1	2043	17.7	11837	47.6	1173	36.4
≥ 4	5804	51.8	12906	78.4	9281	39.8	8363	72.6	8207	33	1586	49.1
**Place of delivery**												
Home	1302	11.6	575	3.5	6135	26.3	900	7.8	6027	24.2	930	28.8

Health Facility	9898	88.3	15894	96.5	17182	73.7	10611	92.2	18842	75.7	2298	71.2
**Delivery assistance**												
Health professional	9138	81.8	14722	89.5	14284	61.8	9091	79.5	14354	58.1	2250	71
Traditional birth attendant	780	6.9	352	2.1	3704	16	403	3.5	3043	12.3	350	11
Other untrained	1246	11.1	1375	8.3	5133	22.2	1938	16.9	7322	29.6	569	18
**Mode of delivery**												
Non-Caesarian section	9267	82.7	9916	60.2	19969	85.6	8990	78.1	22195	89.3	2713	84
Caesarean section	1933	17.2	6553	39.8	3348	14.4	2521	21.9	2674	10.7	515	16
Environmental factors												
**Source of drinking water**												
Improved	9128	81.5	13859	84.2	20248	86.8	9499	82.52	21073	84.7	2530	78.4
Not Improved	2072	18.5	2610	15.8	3069	13.1	2012	17.48	3797	15.3	698	21.6
**Type of Sanitation**												
Improved	7034	67.6	9277	62.4	8175	37.7	6672	64.1	9343	41.3	1979	62.6
Not improved	275	2.6	223	1.5	322	1.5	99	0.9	224	0.9	884	28
Open defecation	3096	29.7	5378	36.1	13162	60.8	3639	34.9	13058	57.7	296	9.4

Community level factors												
**Type of place of residence**												
Urban	3759	33.5	6652	40.3	3806	16.3	4930	42.8	5541	22.2	457	14.2
Rural	7441	66.4	9816	59.6	19512	83.7	6581	57.1	19328	77.7	2771	85.8

N† = weighted counts

Others‡ = Includes Jews, Parsis/Zoroastrians, those following “other” religions, and those with no religion

Supplementary Table 2 - Prevalence of infant and young child feeding (IYCF) indicators among children aged 0-23 months in regional India, NFHS-4

	North		South		East		West		Central		North-East	
	N†	Prevalence‡ (95% CI)	*N*	Prevalence (95% CI)	*N*	Prevalence (95% CI)	*N*	Prevalence (95% CI)	*N*	Prevalence (95% CI)	*N*	Prevalence (95% CI)
**Early initiation of breastfeeding**												
No	7425	66.3 (65.1, 67.5)	8056	48.9 (47.4, 50.5)	13185	56.6 (55.3, 57.7)	5159	44.8 (42.7, 46.9)	17286	69.5 (68.7, 70.3)	1148	35.6 (33.6, 37.5)
Yes	3775	33.7 (32.5, 34.9)	8412	51.1 (49.5, 52.6)	10132	43.5 (42.2, 44.6)	6353	55.2 (53.1, 57.3)	7584	30.5 (29.7, 31.3)	2080	64.4 (62.5, 66.3)
**Exclusive breastfeeding**												
No	1201	43.9 (41.3, 46.1)	1534	42.1(39.6, 44.7)	2257	43.6 (41.6, 45.6)	1121	43.6 (39.7, 47.5)	3073	49.5 (48.1, 50.9)	310	38.4 (35.7, 41.1)
Yes	1530	56.0 (53.9, 58.1)	2107	57.8 (55.2, 60.4)	2919	56.4 (54.4, 58.3)	1451	56.4 (52.5, 60.3)	3133	50.5 (49.0, 51.9)	496	61.6 (58.8, 64.3)
**Predominant Breastfeeding**												
No	2133	78.1 (76.3, 79.7)	3151	86.5 (84.6, 88.2)	4305	83.2 (81.8, 84.5)	2039	79.3 (76.2, 82.1)	4709	75.8 (74.6, 77.1)	707	87.7 (85.7, 89.4)

Yes	598	21.9 (20.2, 23.7)	490	13.5 (11.7, 15.4)	871	16.8 (15.5, 18.2)	533	20.7 (17.9, 23.8)	1497	24.1 (22.9, 25.4)	99	12.3 (10.5, 14.3)
**Bottle feeding**												
No	8879	79.3 (78.3, 80.2)	12563	76.3 (75.1, 77.4)	19593	84.0 (83.2, 84.8)	9679	84.1 (82.2, 85.8)	19417	78.1 (77.4, 78.7)	2734	84.8 (83.6, 85.7)
Yes	2321	20.7 (19.8, 21.6)	3906	23.7 (22.6, 24.8)	3724	16.0 (15.2, 16.8)	1832	15.9 (14.2, 17.8)	5453	21.9 (21.2, 22.6)	494	15.3 (14.3, 16.4)
**Continued breastfeeding at one year**												
No	291	15.5 (13.8, 17.3)	586	20.3 (17.7, 23.1)	335	8.2 (7.1, 9.3)	288	14.5 (11.9, 17.7)	572	14.0 (12.8, 15.3)	43	7.2 (5.9, 8.9)
Yes	1586	84.5 (82.7, 86.2)	2307	79.7 (76.9, 82.3)	3759	91.8 (90.6, 92.9)	1689	85.5 (82.3, 88.1)	3503	85.9 (84.7, 87.1)	552	92.7 (91.1, 94.1)
**Continued breastfeeding at two years**												
No	486	29.9 (27.5, 32.5)	1249	46.8 (43.5, 50.2)	572	15.1 (13.6, 16.8)	658	35.9 (31.8, 40.4)	861	24.2 (22.7, 25.8)	67	13.2 (11.2, 15.5)
Yes	1137	70.1 (67.5, 72.5)	1419	53.2 (49.8, 56.5)	3206	84.9 (83.2, 86.4)	1170	64.0 (59.6, 68.2)	2699	75.8 (74.3, 77.3)	438	86.7 (84.4, 88.8)
**Children ever breastfed**												
No	323	2.9 (2.5, 3.2)	718	4.4 (3.7, 5.1)	511	2.2 (1.9, 2.4)	289	2.5 (1.9, 3.1)	604	2.4 (2.2, 2.6)	115	3.6 (2.8, 4.4)
Yes	10877	97.1 (96.7, 97.4)	15751	95.6 (94.9, 96.2)	22806	97.8 (97.5, 98.1)	11223	97.5 (96.8, 98.0)	242625	97.5 (97.3, 97.8)	3113	96.4 (95.5, 97.1)

**Introduction of solid, semi-solid or soft foods**												
No	1042	62.4 (59.2, 65.5)	866	39.3 (35.9, 42.7)	1897	57.3 (54.7, 59.7)	849	52.4 (47.5, 57.3)	2226	61.8 (60.0, 63.5)	178	48.1 (44.2, 52.0)
Yes	627	37.5 (34.5, 40.7)	1339	60.7 (57.3, 64.1)	1417	42.7 (40.3, 45.2)	772	47.6 (42.7, 52.5)	1376	38.2 (36.5, 40.0)	193	51.9 (48.0, 55.8)

N† = Weighted total number of children aged 0-23 months within each IYCF indicators; 95% CI: 95% confidence interval.

Prevalence‡ = represents the overall weighted proportion of children with diarrhoea for each level (‘No’, ‘Yes’) of infant and young child feeding indicators.

- Early initiation of breastfeeding was defined as the proportion of children within 0 – 23 months of age who were breastfed within one hour of birth.
- Exclusive breastfeeding was defined as the proportion of infants 0–5 months of age who received breast milk as the only source of nourishment but allowed oral rehydration solution, drops or syrups of vitamins and medicines.
- Predominant breastfeeding was defined as the proportion of infants 0–5 months of age who received breast milk as the main source of nourishment but allowed water, water-based drinks, fruit juice, oral rehydration solution, drops or syrups of vitamins and medicines.
- Bottle feeding was defined as the proportion of children 0–23 months of age who were fed with a bottle during the previous day.
- Continued breastfeeding at 1 year was defined as the proportion of children 12–15 months of age who were fed breast milk.
- Continued breastfeeding at 2 years was defined as the proportion of children 20–23 months of age who were fed breast milk.
- Children ever breastfed was defined as the proportion of children born in the last 24 months who were ever breastfed.
- Introduction of solid, semi-solid or soft foods was defined as the proportion of infants 6–8 months of age who received solid, semi-solid or soft foods.

Supplementary Table 3 – Association between and infant and young child feeding (IYCF) indicators and diarrhoea in regional India, NFHS-4

	North		South		East		West		Central		North-East		National estimate	
	Unadjusted OR† (95% CI)	P value	Unadjusted OR (95% CI)	P value	Unadjusted OR (95% CI)	P value	Unadjusted OR (95% CI)	P value	Unadjusted OR (95% CI)	P value	Unadjusted OR (95% CI)	P value	Unadjusted OR (95% CI)	P value
**Early initiation of breastfeeding**														
No	1.00		1.00		1.00		1.00		1.00		1.00		1.00	
Yes	0.80 (0.70, 0.92)	0.002	0.75 (0.63, 0.90)	0.003	0.83 (0.74, 0.93)	0.002	0.84 (0.67, 1.05)	0.144	0.76 (0.70, 0.82)	<0.001	0.55 (0.43, 0.70)	<0.001	0.71 (0.67, 0.75)	<0.001
**Exclusive breastfeeding**														
No	1.00		1.00		1.00		1.00		1.00		1.00		1.00	
Yes	0.57 (0.13, 0.19)	<0.001	0.75 (0.48, 1.17)	0.217	0.69 (0.54, 0.88)	0.003	0.69 (0.08, 1.11)	0.134	0.57 (0.49, 0.67)	<0.001	0.56 (0.32, 0.99)	0.047	0.60 (0.53, 0.67)	<0.001
**Predominant breastfeeding**														
No	1.00		1.00		1.00		1.00		1.00		1.00		1.00	
Yes	1.21 (0.87, 1.67)	0.251	1.17 (0.63, 2.18)	0.601	1.22 (0.91, 1.64)	0.176	1.51 (0.86, 2.66)	0.149	1.38 (1.17, 1.63)	<0.001	1.33 (0.73, 2.42)	0.339	1.45 (1.27, 1.65)	<0.001
**Bottle feeding**														
No	1.00		1.00		1.00		1.00		1.00		1.00		1.00	
Yes	1.37 (1.17, 1.60)	<0.001	1.23 (1.01, 1.49)	0.033	1.40 (1.22, 1.60)	<0.001	1.23 (0.92, 1.65)	0.147	1.50 (1.38, 1.63)	<0.001	1.19 (0.90, 1.58)	0.217	1.40 (1.31, 1.49)	<0.001
**Continued breastfeeding at one year**														
No	1.00		1.00		1.00		1.00		1.00		1.00		1.00	
Yes	1.13 (0.70, 1.82)	0.599	0.58 (0.35, 0.96)	0.035	1.09 (0.72, 1.64)	0.682	0.92 (0.45, 1.90)	0.842	0.98 (0.78, 1.24)	0.913	1.50 (0.72, 3.09)	0.27	0.93 (0.77, 1.12)	0.477
**Continued breastfeeding at two years**														
No	1.00		1.00		1.00		1.00		1.00		1.00		1.00	

Yes	1.47 (0.96, 2.25)	0.074	1.28 (0.83, 1.98)	0.253	0.84 (0.57, 1.23)	0.381	2.26 (1.26, 4.07)	0.006	0.93 (0.75, 1.15)	0.509	0.43 (0.23, 0.80)	0.008	1.18 (1.01, 1.38)	0.037
**Children ever breastfed**														
No	1.00		1.00		1.00		1.00		1.00		1.00		1.00	
Yes	1.06 (0.71, 1.59)	0.746	0.87 (0.58, 1.31)	0.516	1.12 (0.74, 1.70)	0.562	1.31 (0.74, 2.33)	0.34	0.95 (0.77, 1.17)	0.656	0.59 (0.28, 1.24)	0.169	1.06 (0.90, 1.24)	0.442
**Introduction of solid, semi-solid or soft foods**														
No	1.00		1.00		1.00		1.00		1.00		1.00		1.00	
Yes	1.29 (0.96, 1.75)	0.088	1.21 (0.75, 1.94)	0.418	0.85 (0.64, 1.12)	0.267	0.96 (0.60, 1.56)	0.896	1.36 (1.14, 1.62)	<0.001	0.97 (0.50, 1.88)	0.947	1.06 (0.92, 1.21)	0.392

Unadjusted OR† = Unadjusted odds ratio; 95% CI: 95% confidence interval**.**

- Early initiation of breastfeeding was defined as the proportion of children within 0 – 23 months of age who were breastfed within one hour of birth.
- Exclusive breastfeeding was defined as the proportion of infants 0–5 months of age who received breast milk as the only source of nourishment but allowed oral rehydration solution, drops or syrups of vitamins and medicines.
- Predominant breastfeeding was defined as the proportion of infants 0–5 months of age who received breast milk as the main source of nourishment but allowed water, water-based drinks, fruit juice, oral rehydration solution, drops or syrups of vitamins and medicines.
- Bottle feeding was defined as the proportion of children 0–23 months of age who were fed with a bottle during the previous day.
- Continued breastfeeding at 1 year was defined as the proportion of children 12–15 months of age who were fed breast milk.
- Continued breastfeeding at 2 years was defined as the proportion of children 20–23 months of age who were fed breast milk.
- Children ever breastfed was defined as the proportion of children born in the last 24 months who were ever breastfed.
- Introduction of solid, semi-solid or soft foods was defined as the proportion of infants 6–8 months of age who received solid, semi-solid or soft foods.

Supplementary Table 4 - Number of missing information after adjusting for potential confounding and exposure factors

IYCF indicators	Unadjusted	Adjusted[#]	Difference[$]
**Early initiation of breastfeeding**			
North	15630	14239	1391
South	9443	8555	888
East	19813	18113	1700
West	6610	6514	96
Central	29213	26213	3000
North East	13435	12272	1163
**Exclusive Breastfeeding**			
North	3741	3272	469
South	2,115	1712	403
East	4,547	4,038	509
West	1,541	1,519	22
Central	7,471	6,550	921
North East	3,191	2,880	311
**Predominant Breastfeeding**			
North	3,741	3,272	469
South	2,115	1,712	403
East	4,547	4,038	509
West	1,541	1,519	22

Central	7,471	6,550	921
North East	3,191	2,880	311
**Bottle feeding**			
North	15,630	14,239	1391
South	9,443	8,555	888
East	19,813	18,113	1700
West	6,610	6,514	96
Central	29,213	26,213	3000
North East	13,435	12,272	1163
**Continued breastfeeding at one year**			
North	2,673	2,472	201
South	1,640	1,520	120
East	3,435	3,163	272
West	1,186	1,167	19
Central	4,789	4,330	459
North East	2,481	2,269	212
**Continued breastfeeding at two years**			
North	2,278	2,108	170
South	1,453	1,362	91
East	3,206	2,977	229
West	987	977	10
Central	4,180	3,840	340

North East	2,083	1,916	167
**Ever Breastfeeding**			
North	15,630	14,239	1391
South	9,443	8,555	888
East	19,813	18,113	1700
West	6,610	6,514	96
Central	29,213	26,213	3000
North East	13,435	12,272	1163
**Complementary feeding**			
North	2,311	2,104	207
South	1,272	1,156	116
East	2,720	2,475	245
West	914	903	11
Central	4,120	3,667	453
North East	1,728	1,594	134

$ = Number of missing information, # = adjusting for potential confounding and exposure factors
